# Survival status and factors associated with treatment outcome of severely malnourished children admitted to Ayder referral hospital: a cross-sectional study

**DOI:** 10.1186/s40795-017-0186-7

**Published:** 2017-07-25

**Authors:** Mengistu Girma Tirore, Tesfay Mehari Atey, Haftay Berhane Mezgebe

**Affiliations:** 1 Department of Pharmacy, College of Health Sciences, Adigrat University, Adigrat, Tigray Ethiopia; 2 Department of Clinical Pharmacy, School of Pharmacy, College of Health Sciences, Mekelle University, Mekelle, Tigray Ethiopia

**Keywords:** Survival status, Treatment outcome, Severe acute malnutrition

## Abstract

**Background:**

Severe acute malnutrition remains the major cause of morbidity and mortality for children under five years of age in developing countries. The prevalence of wasting, underweight and stunting has remained high in Ethiopia and even unacceptably higher in Tigray region. The objective of the study is to assess the survival status and treatment outcome of patients with severe acute malnutrition and to identify contributing factors for poor treatment outcome.

**Methods:**

An institutional-based cross-sectional study was conducted on 195 patients, selected using systematic random sampling technique, from 24-Mar-2015 to 7-Jun-2015 in Ayder Referral Hospital. Logistic regression was carried out to identify factors associated with treatment outcome. Rates of mortality associated with the disease were determined using Kaplan-Meier survival analysis. A Log Rank, Breslow, and Tarone-Ware test were employed for the overall comparisons of the survival curves. Statistical significance was declared at *p* – value <0.05.

**Result:**

Out of 195 children admitted with SAM, the cure, death, defaulter, non-respondent and transferred-out rates were 22.1%, 3.6%, 43.6%, 9.2% and 21.5% respectively. Overall, 43.6% of the children were recovered from their disease. The mean length of stay of a ‘recovered’ child in the hospital was 21.56 ±1.27 days (95% CI: 19.04–24.09 days). Free from acute febrile illness (AOR = 4.20, 95% CI: 1.10–16.09, *p* < 0.036) and usage of deworming medications (AOR = 0.36, 95% CI: 0.14–0.93, *p* < 0.036) were significantly associated with positive and negative treatment outcomes respectively. Children with >70% of weight for height (WFH) and mid-upper arm circumference (MUAC) of >12 cm at admission had a better treatment outcome than children with WFH of ≤ 70% (*p* < 0.038) and MUAC of ≤ 12 cm (*p* < 0.090). Treatment using ready-to-used therapeutic food (RUTF) provided a longer all-cause mortality protection than the treatment using F-75 and F-100 (*p* < 0.010).

**Conclusion:**

The cure rate in this study was found to be sub-optimal. Absence of acute febrile illness and deworming medication use were factors contributing to good treatment outcome. A WFH of >70%, MUAC of ≥ 12 cm and treatment using RUTF provided a longer all-cause mortality protection.

## Background

Although abundant food is available worldwide, the international Federation of Red Cross and Red Crescent Societies (IFRC) 2011 report claimed that every night one in six persons sleep without having food. Besides this, this report also proved that millions of young children agonize from the ominous effects of undernutrition [1].

Malnutrition remains to be a wide-reaching problem despite significant improvements in prevention and treatment of the disease. Many studies proved the dreadful impact of undernutrition on the life of children. On the global scale, approximately 9 million newborns untimely lose their life before reaching the age of five days, of which 30% of these premature deaths are attributable to undernutrition [2]. About 178 million and 55 million children under five years are also affected by stunting and wasting secondary to undernutrition respectively [3, 4].

The World Health Organization (WHO) and United Nations Children’s Emergency Fund (UNICEF) defined severe acute malnutrition (SAM) as a very low weight for height (WFH) less than (−) 3 standard deviations below the median reference population or WFH ratio of below 70%, visible severe wasting or presence of nutritional edema (pitting edema) or a mid-upper arm circumference (MUAC) less than 11 cm (cm) [5, 6]. SAM remains the top-killer disease for children under five years of age. This disease affects 20 million of these populations and is responsible for more than 1 million annual deaths worldwide [7, 8]. Furthermore, child with SAM has a ninefold more mortality rates than a well-nourished child. In developing countries, 20–30% and 50–60% case fatality rates of SAM are accounted to marasmus and kwashiorkor respectively [8–10].

As a developing country with an elongated history of food and dietetic insecurity, enormous percentage of the Ethiopian population has been affected by uninterrupted famines. The subsistence of children and women in Ethiopia are at a greater risk because of the tremendously high level of malnutrition even during the virtuous non-drought seasons. According to the Ethiopian Demographic Health Survey (DHS) report of 2011, the prevalence of wasting, underweight and stunting in Ethiopia was 10%, 29% and 44%, and in Tigray, 10.3%, 35.1% and 51.4% respectively [11].

In retort to the high malnourishment degree in Ethiopia, UNICEF, World Food Program (WFP), Ministry of Health (MOH) and Disaster Prevention and Preparedness Commission (DPPC)) launched Enhanced Outreach Strategy Program all over the country and this program has been operationalized in Tigray since 2005 [7]. Despite the launching of some programs to curtail the dire consequences of SAM, no studies related to survival experience and treatment outcome of SAM was not conducted in Ayder Referral Hospital (ARH). Assessment of the treatment outcome and survival experience of children with SAM, and identifying contributing factors for poor treatment outcomes are vital to the proper management of the disease.

## Methods

An institutional-based cross-sectional study was conducted from 24-Mar-2015 to 7-Jun-2015 in ARH. All children with SAM admitted to ARH were considered as the source population whereas all children with SAM admitted to ARH during the study period were considered as the study population. Children with SAM who were admitted to the hospital during the study period, aged 6–59 months, and whose parents or care givers were willing to give informed consent were included in the study.

On average, 780 children with SAM were admitted to the hospital during the study period (statistics office of the ARH). The smaple size was calculated to be 195 considering 1.96 for the standard normal variable (Z-value) with 5% level of significance (α-value), 80% power of study (β-value), 95% confidence interval (CI), 5% margin of error, 0.87 prevalence [11] and 10% contingency. A sampling interval (k) was calculated to be four (k = 780/195 = 4). A systematic random sampling technique was employed to select samples from the study population.

Data collection tool was developed to collect patients’ demographic and clinical information, anthropometric measurements, treatment phases, types of therapeutic foods, types and numbers of prescribed medications and status of treatment outcome from the patient’s medical charts, treatment charts, and laboratory data reports. The data were collected by five trained data collectors (three nurses and two pharmacists). Moreover, the data collection tool was pre-tested in 5% of the sample size (i.e., 10 patients).

In this study, a child with SAM was considered to be “recovered” from the disease when the status of the treatment outcome was categorized either as “cured” and/or “transferred out” to other health institutions due to improvements in the health status of the patient. On the contrary, a child with SAM was operationally defined as “censored” when the patient’s clinical condition after the treatment was either recorded as “death”, “defaulters” and “non-respondent”.

The collected data were cleaned, coded and fed into Statistical Package for Social Sciences (SPSS) for Windows version 21 (SPSS Inc., Chicago, IL, USA). In order to summarize the patients’ socio-demographic and clinical characteristics, descriptive statistics such as frequency, percentage, mean and standard deviation were employed. The relationship between treatment outcome and independent variables was computed by logistic regression analysis. The survival analysis was carried out using Kaplan-Meier method. A Log Rank (Mantel-Cox), Breslow (Generalized Wilcoxon) and Tarone-Ware tests were employed for the overall comparisons of the survival curves. Factors were identified as statistically significant when *p* - value <0.05 at 95% confidence interval [CI].

## Results

**Fig. 1:**
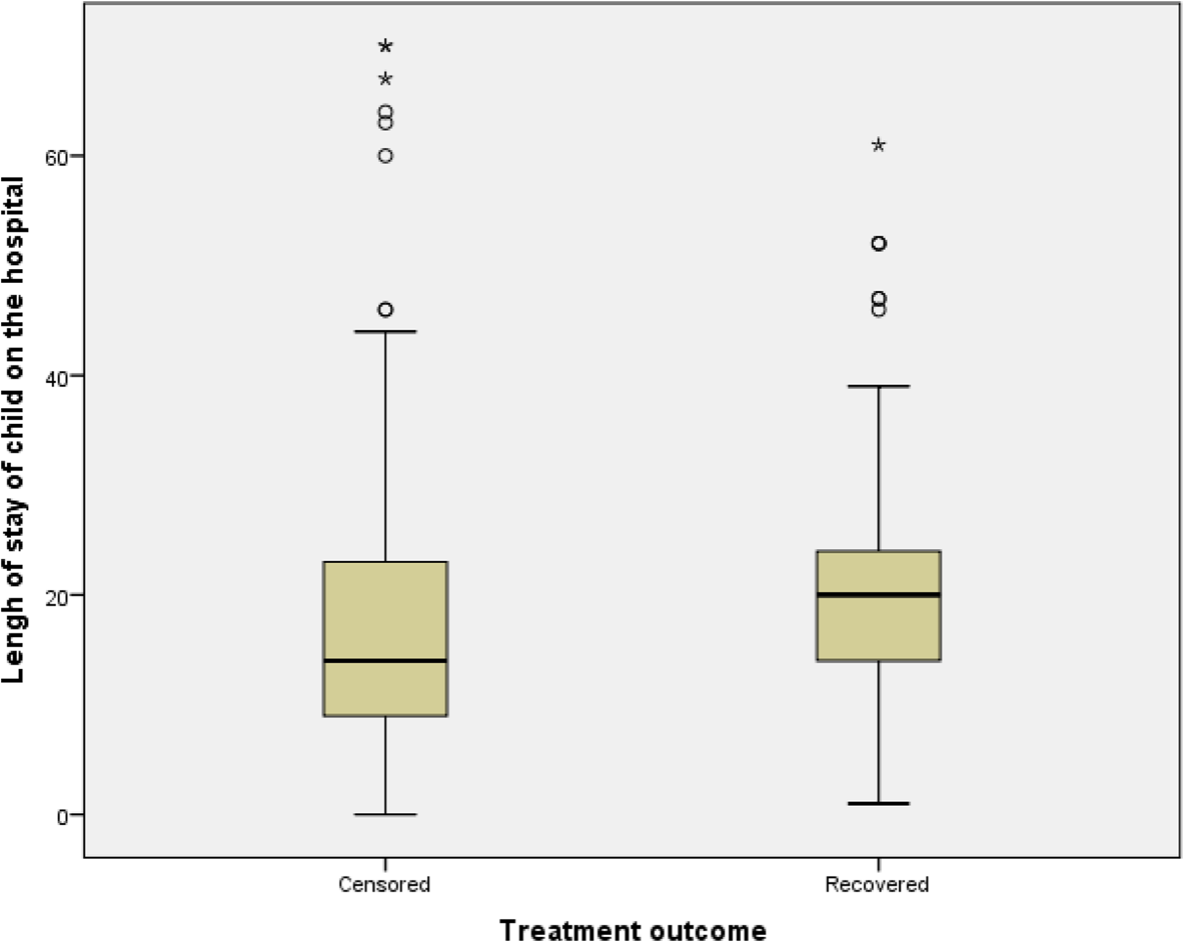
Box-and-whisker plots of treatment outcome in relation to the length of stay for children with severe acute malnutrition in Ayder Referral Hospital, 2015, *n* = 195

From a total of 195 children diagnosed with SAM, almost half (50.8%) of them were males and about three-fourth (67.2%) of the participants’ age was 13 to 59 months old. Additionally, the mean length of stay of a ‘censored’ child and a ‘recovered’ child in the hospital were 18.22 days (SE: ±1.44 days; 95% CI: 15.37 to 21.07 days) and 21.56 days (SE: ±1.27 days; 95% CI: 19.04 to 24.09 days) respectively (Fig. 1).

**Fig. 2:**
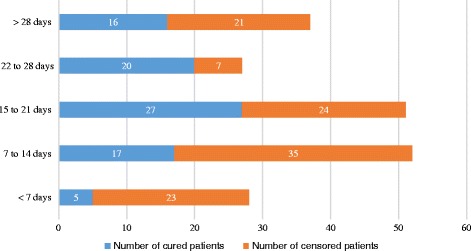
Comparison of the number of cured and censored patients according to the length of admission of children with severe acute malnutrition in Ayder Referral Hospital, 2015, *n* = 195

In the present study, it was shown that increased number of censored patients was correlated with a shorter stay in the hospital unlike the longer the stay in the hospital, the greater number of cured patients (Fig. 2).

The anthropometric conditions of children with SAM are summarized in Table 1. The percentage of children with MUAC of <11 cm almost halved from 60% at admission to 30.7% at discharge. Similarly, WFH measurement indicated that the number of children with WFH of 40–70% decreased significantly from 50% at admission to 29.7% at discharge. Severe edema (+++ edema) was also decreased significantly from 11.8% at admission to 1.0% at discharge. Hospital card record of the patients at admission also showed that over half of children with SAM had a MUAC of 6 to 10.9 cm (*n* = 117, 60%), WFH of 40–70% (*n* = 96, 50.0%) and no edema (*n* = 116, 59.5%) (Table 1).

**Table 1:** Anthropometric measurements and edematous conditions of children with severe acute malnutrition at admission and discharge in Ayder Referral Hospital, 2015, *n* = 195

Anthropometry	At admission, N (%)	At discharge, N (%)
MUAC (Mid Upper Arm Circumstance)		
> 12 cm	5 (2.6)	5 (2.8)
11–12 cm	73 (37.4)	119 (66.5)
< 11 cm	117 (60.0)	55 (30.7)
WFH (Weight for Height)		
≥ 85%	9 (4.7)	9 (5.1)
80–84%	19 (9.9)	55 (31.4)
71–79%	68 (35.4)	59 (33.7)
≤ 70%	96 (50.0)	52 (29.7)
Edema		
No edema	116 (59.5)	181 (92.8)
(+) edema	27 (13.8)	8 (4.1)
(++) edema	29 (14.9)	4 (2.1)
(+++) edema	23 (11.8)	2 (1.0)

**Fig. 3:**
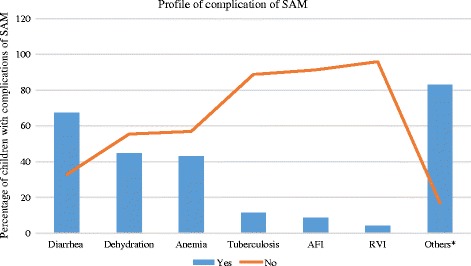
Profile of complications of severe acute malnutrition among children attending Ayder Referral Hospital, 2015, *n* = 195

Regarding medical complications of the SAM, about one-third (67.2%) of the study participants had diarrhea as a complication of SAM followed by dehydration (44.6%) and anemia (43.1%) (Fig. 3).

### Treatment phases, therapeutic feeding and routine medications

Majority of the patients were given F-75 (92.8%), F-100 (62.0%), RUTF (58.4%) in the phase 1, transition phase and phase 2 respectively. The most common types of prescribed medications were antibiotics which accounted for 97.4% of the medications followed by folic acid and vitamin A that accounted for 64.1% and 51.8% respectively (Table 2).

**Table 2:** Profiles for phases of treatment, types of therapeutic feeding and types of routine medications for children with severe acute malnutrition admitted to Ayder Referral Hospital, 2015, *n* = 195

Variables		N (%)
Treatment phase		
Phase 1 (*n* = 193)	F75	181 (92.8)
	F100	12 (6.2)
Transition phase (*n* = 150)	F75	47 (31.3)
	F100	93 (62.0)
	RUTF	10 (6.7)
Phase 2 (*n* = 101)	F100	42 (41.6)
	RUTF	59 (58.4)
Medications		
Antibiotic	Yes	190 (97.4)
	No	5 (2.6)
Vitamin A	Yes	101 (51.8)
	No	94 (48.2)
Folic acid	Yes	125 (64.1)
	No	70 (35.9)
Zinc	Yes	18 (9.2)
	No	177 (90.8)
Deworming medications	Yes	26 (13.3)
	No	169 (86.7)
ReSoMal	Yes	96 (49.2)
	No	99 (50.8)

*Abbreviations*: *ReSoMal* Rehydration solution for malnutrition, *RUTF* Ready to use therapeutic food

### Treatment outcome and associated factors

**Fig. 4:**
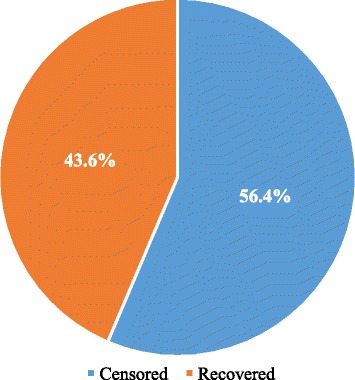
Overall status of treatment outcome for children with severe acute malnutrition admitted to Ayder Referral Hospital, 2015, *n* = 195

Regarding the overall status of treatment outcome of children with SAM, 43.6% (*n* = 85) of the patients were recovered from their disease compared to 56.4% (*n* = 110) of the patients whose treatment was censored. In addition to this, 22.1%, 3.6%, 43.6%, 9.2% and 21.5% of the patients were cured, died, defaulting their treatment, non-respondent to their treatment and transferred out to a nearby health centres for continuation of their management respectively (Table 3 and Fig. 4).

**Table 3:** Components of treatment outcome for children with severe acute malnutrition admitted to Ayder Referral Hospital, 2015, *n* = 195

Treatment outcome	N (%)
Recovered	
Cured	43 (22.1%)
Transferred-out	42 (21.5%)
Total	85 (43.6%)
Censored	
Defaulter	85 (43.6%)
Non-respondent	18 (9.2%)
Died	7 (3.6%)
Total	110 (56.4%)

The results of multivariate analyses showed that two factors were found to be significantly associated with treatment outcome after incorporating variables which were significant at *p* < 0.2 in the univariate analysis into the multivariate analyses. Accordingly, the odds of being recovered from SAM for patients without a complication of acute febrile illness were almost fourth-fold (AOR = 4.20, 95% CI: 1.10–16.09, *p* < 0.036) more compared to patients with this complication. In contrary, patients who were not routinely using deworming medications were having 64% (AOR = 0.36, 95% CI: 0.14–0.93, *p* < 0.036) lower odds of recovering from SAM compared to patients who were routinely using deworming medications (Table 4).

**Table 4:** Results of univariate and multivariate logistic regression analysis for factors affecting treatment outcome of children with severe acute malnutrition admitted to Ayder Referral Hospital, 2015, *n* = 195

Variable	Treatment Outcome		*p* – value	COR	*p* – value	AOR
	Censored, n (%)	Recovered, n (%)				
Admission status						
Referred	66 (59.5)	45 (40.5)		1.00		1.00
New admission	41 (55.4)	33 (44.6)	0.584	1.18 (0.65–2.14)	0.808	1.08 (0.56–2.13)
Re admission	3 (30.0)	7 (70.0)	0.086	3.42 (0.84–13.94)	0.078	4.04 (0.86–19.03)
WFH classification						
> 85%	2 (22.2)	7 (77.8)		1.00		1.00
80–84%	7 (36.8)	12 (63.2)	0.444	0.49 (0.08–3.05)	0.721	0.69 (0.09–5.25)
71–79%	39 (57.4)	29 (42.6)	0.065	0.21 (0.04–1.11)	0.277	0.35 (0.05–2.32)
< 70%	60 (62.5)	36 (37.5)	0.033	0.17 (0.03–0.87)	0.175	0.27 (0.04–1.78)
MUAC classifications						
> 12 cm	1 (20.0)	4 (80.0)		1.00		1.00
11–12 cm	37 (50.7)	36 (49.3)	0.216	0.24 (0.03–2.28)	0.915	0.87 (0.07–11.16)
< 11 cm	72 (61.5)	45 (38.5)	0.102	0.16 (0.02–1.44)	0.611	0.52 (0.04–6.68)
Presence of complication of tuberculosis						
Yes	16 (72.7)	6 (27.3)		1.00		1.00
No	94 (54.3)	79 (45.7)	0.108	2.24 (0.84–6.00)	0.172	2.14 (0.72–6.35)
Presence of complication of acute febrile illness						
Yes	14 (82.4)	3 (17.6)		1.00		1.00
No	96 (53.9)	82 (46.1)	0.034	3.97 (1.12–14.36)	0.036	4.20 (1.10–16.09) *
Presence of complication of retroviral infection						
Yes	7 (87.5)	1 (12.5)		1.00		1.00
No	103 (55.1)	84 (44.9)	0.106	5.71 (0.69–47.32)	0.149	4.90 (0.56–42.47)
Usage of routine deworming medications						
Yes	11 (42.3)	15 (57.7)		1.00		1.00
No	99 (58.6)	70 (41.4)	0.124	0.52 (0.23–1.20)	0.036	0.36 (0.14–0.93) *

*AOR* adjusted odds ratio, *COR* crude odds ratio, *MUAC* middle upper arm circumference, *WFH* weight for height

*Statistically significant at *p* < 0.05

### Mortality rates and survival analyses

**Fig. 5:**
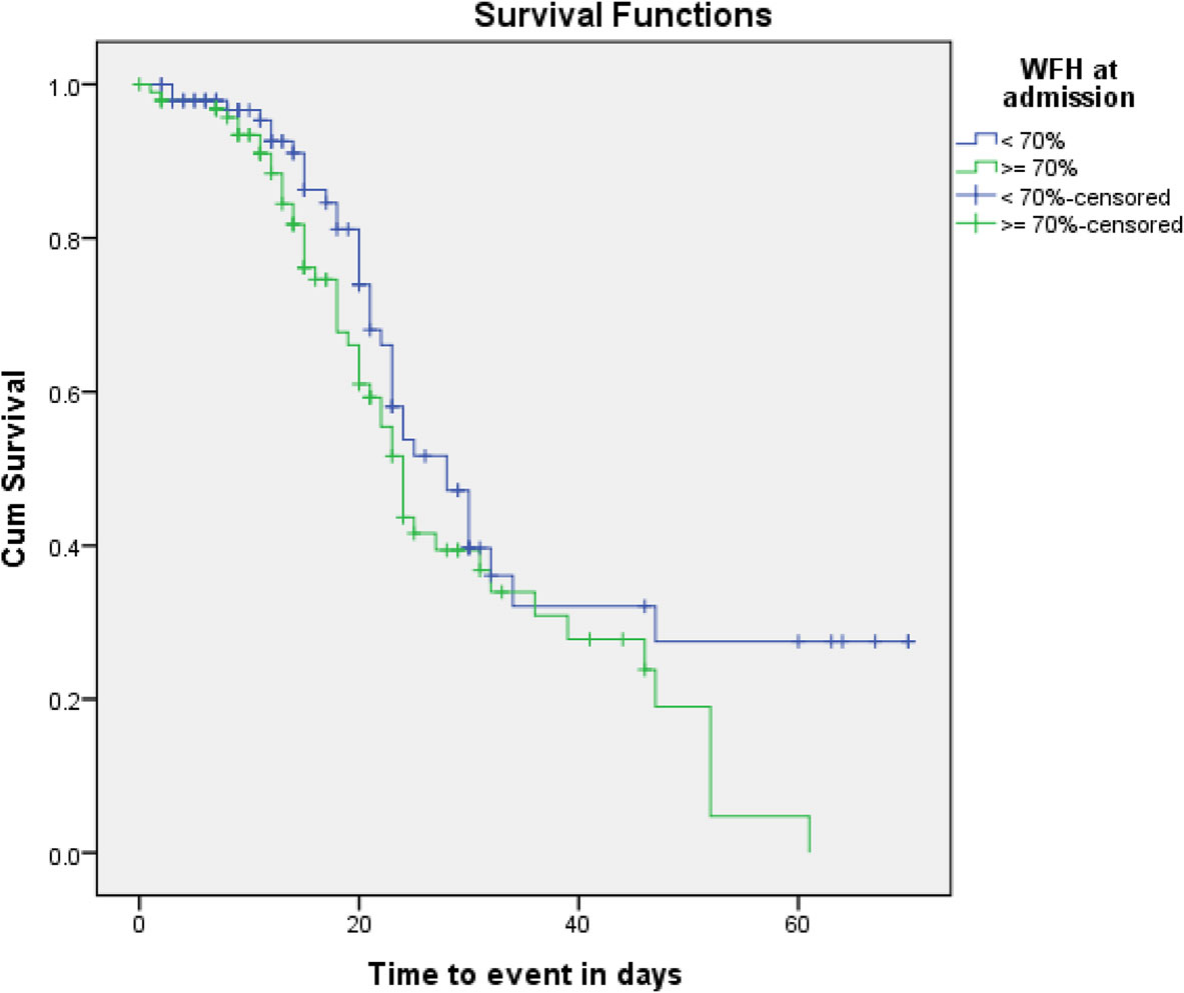
Kaplan-Meier survival curve of treatment outcome for children with severe acute malnutrition by weight for height at admission in Ayder Referral Hospital, 2015, *n* = 195

The KM survival curve for WFH at admission illustrated that children with >70% of WFH at admission had a better treatment outcome than children with WFH of ≤ 70% at admission (*p* < 0.038). The test of equality of survival distributions for the different levels of WFH showed Chi-Square results of 7.68, 8.45 and 7.92 for the Log Rank (Mantel-Cox) (*p* < 0.053), Breslow (Generalized Wilcoxon) (*p* < 0.038) and Tarone-Ware (*p* < 0.048) respectively (Fig. 5).

**Fig. 6:**
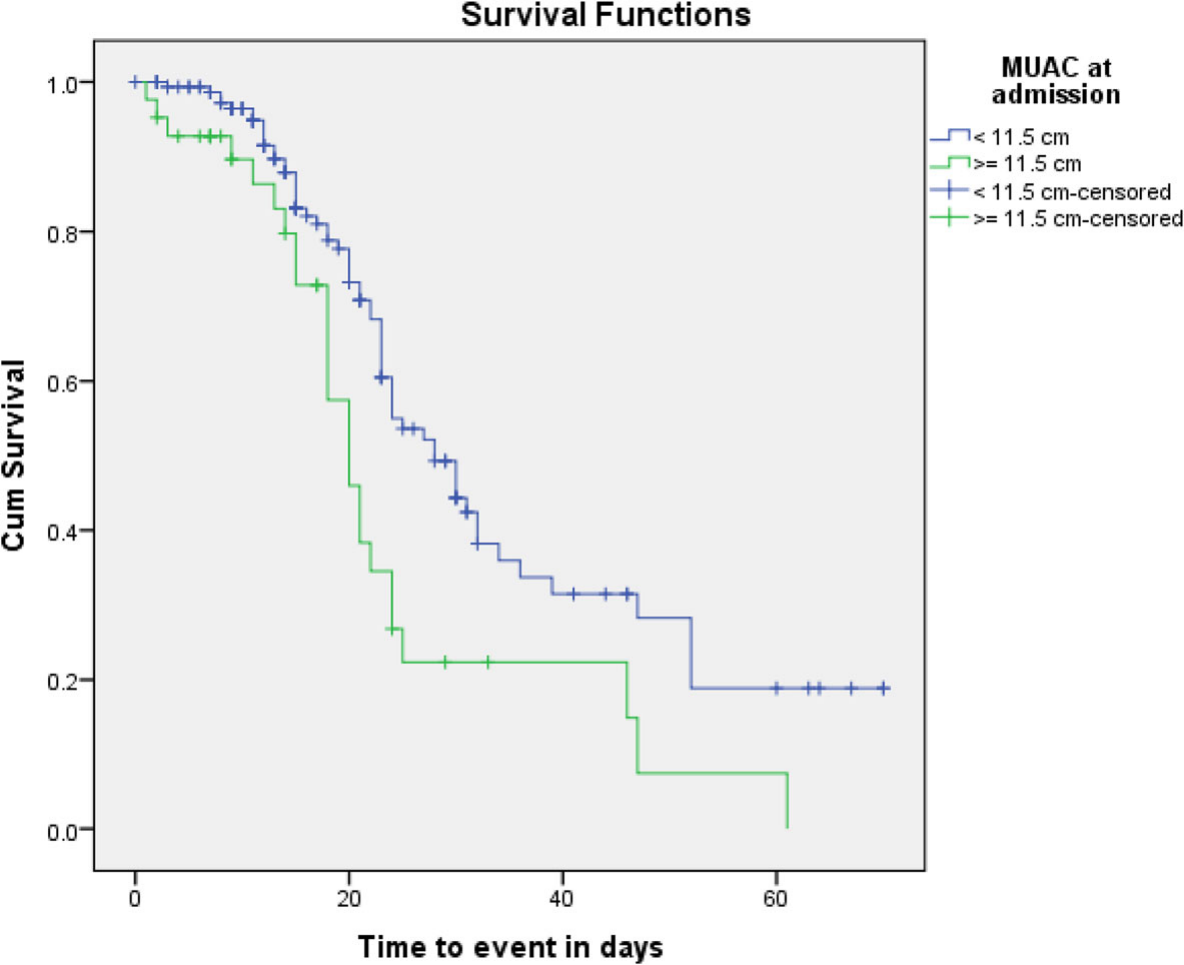
Kaplan-Meier survival curve of treatment outcome for children with severe acute malnutrition by mid upper arm circumference at admission in Ayder Referral Hospital, 2015, *n* = 2015

The KM survival curve for MUAC at admission in relation to time to event illustrated that those children with MUAC value of >12 cm at admission had better treatment outcome compared to those who had MUAC of ≤ 12 cm at admission (*p* < 0.090). The test of equality of survival distributions for the different levels of MUAC at admission showed Chi-Square results of 7.17, 9.27 and 8.77 for the Log Rank (Mantel-Cox) (*p* < 0.028), Breslow (Generalized Wilcoxon) (*p* < 0.010) and Tarone-Ware (*p* < 0.012) respectively (Fig. 6).

A comparison of survival curves for the types of therapeutic foods in the transition phase also revealed that treatment using RUTF provided a longer all-cause mortality protection than the treatment using F-75 and F-100 (*p* < 0.010). The test of equality of survival distributions for the different levels of therapeutic foods at transition phase showed Chi-Square results of 4.81, 2.99 and 3.94 for the Log Rank (Mantel-Cox) (*p* < 0.028), Breslow (Generalized Wilcoxon) (*p* < 0.010) and Tarone-Ware (*p* < 0.012) respectively.

## Discussions

In this study, the average length of hospital stay was found to be 19.68 ± 13.8 days which was lower than reports from studies conducted in other African countries (a length of stay varying from 28 to 35 days). This finding indicated that the average length of stay in this setting was relatively shorter than reports from 13 African countries [12]. This could be attributable to differences in the health institution setting where the study setting was referral hospital and supposed to transfer-out recovered patients towards nearby health institutions. Moreover, the current study showed that antibiotics were the most commonly prescribed medications which was in line with other studies that indicated the inclusion of antibiotics for children with SAM [13]. These patients are at risk of severe infections and hence antibiotics could be prescribed customarily as part of their nutritional therapy.

The cure rate, death rate, defaulting rate, non-respondent rate and transferred out rate was found to be 22.1%, 3.6%, 43.6%, 9.2% and 21.5% respectively. These results are not in line with a study conducted in Southern Ethiopia that revealed87% cure rate, 3.6% death rate, 9.1% defaulting rate and 0.3% non-respondent rate [13]. Therefore, cure rate in the present study was lower, defaulter and non-respondent rate were higher compared to the aforementioned finding. Despite there is no clear justification of these discrepancies, appropriate utilization of the protocol for management of SAM can maximize the cure rate and minimize unwanted outcomes of treatment.

The cure rate, unlike the death rate, was found to be less than the standard criteria as per the SAM management protocol [14]. According to this standard, the death rate is acceptable but the cure rate is not acceptable that could be attributable to the relatively longer admission period (approximately 20 days), low nutritional value of the commonly ingested foods, and economic constraints of the community. This finding was congruent with a study done in Gondor University (Ethiopia) [15]. Another study conducted in Tigray(Ethiopia) also demonstrated that lack of complementary foods was important predictor of a child undernutrition and chronic malnutrition was a public health problem in the study community [16].

The results of multivariate analyses depicted that complication of acute febrile illness and usage of deworming medications were significantly associated with poor and good treatment outcomes respectively. Children without the complication of acute febrile illness were more likely to recover from SAM compared to children with this complication. Patients with acute febrile illness would further compound the disease and complicate the SAM. This, in turn, could negatively affect the treatment outcome. In contrary, patients who were not using deworming medications, as part of their management modality, were less likely to recover from SAM compared to patients who were using these medications. The use of deworming medications could reduce gastrointestinal related infections which, in turn, maintain the integrity of the gastrointestinal tract and hasten absorption of nutrients and thereby recovery from SAM disease.

The KM survival analyses and mortality rates of SAM showed that having WFH of >70% and MUAC of ≥ 12 cm at admission were important predictors of better treatment outcome. This finding corresponded with studies done in Northern and Southern Ethiopia [17, 18]. These measurements help identify children who may be wasted or severely wasted. This wasting is usually caused by a recent illness or food shortage that causes acute and severe weight loss [19]. Another finding revealed in this study was that treatment using RUTF provided a longer all-cause mortality protection than treatment using F-75 and F-100. This might be attributable to the faster recovery rate associated with RUTF and higher acceptability of RUTF than F-75 and F-100 [20].

The study had certain limitations. Factors associated with treatment outcome could have been preponderantly identified using a longitudinal research designs than the cross-sectional nature of the present study. The study was also conducted in a single centre which could limit the generalizability of the findings to a broader milieu.

## Conclusions

In general, the cure rate for children with SAM in the tertiary referral hospital was found to be sub-optimal. Deworming medications use was significantly associated with good treatment outcome unlike the association of poor treatment outcome with the presence of acute febrile illness. Having WFH of >70% and MUAC of ≥ 12 cm at the admission were important predictors of better treatment outcome and treatment using RUTF provided a longer all-cause mortality protection than F-75 and F-100. Consequently, due emphasis should be given in improving early detection and treatment of severely malnourished children.

## Abbreviations

AOR: adjusted odds ratio
CI: confidence interval
Cm: centimetre
COR: crude odds ratio
CSA: Central Statistics Authority
DHS: Demographic Health Survey
DPPC: Disaster Prevention and Preparedness Commission
MUAC: middle upper arm circumference
SAM: severe acute malnutrition
UNICEF: United Nations Children’s Emergency Fund
WFH: weight for height
WHO: World Health Organizations.
